# Autocrine signaling can explain the emergence of Allee effects in cancer cell populations

**DOI:** 10.1371/journal.pcbi.1009844

**Published:** 2022-03-03

**Authors:** Philip Gerlee, Philipp M. Altrock, Adam Malik, Cecilia Krona, Sven Nelander

**Affiliations:** 1 Mathematical Sciences, Chalmers University of Technology, Gothenburg, Sweden; 2 Mathematical Sciences, University of Gothenburg, Gothenburg, Sweden; 3 Department of Integrated Mathematical Oncology, Moffitt Cancer Center, Tampa, Florida, United States of America; 4 Department of Evolutionary Theory, Max Planck Institute for Evolutionary Biology, Plön, Germany; 5 Department of Immunology, Genetics and Pathology, Uppsala University, Uppsala, Sweden; Leiden University Faculty of Science: Universiteit Leiden Faculteit der Wiskunde en Natuurwetenschappen, NETHERLANDS

## Abstract

In many human cancers, the rate of cell growth depends crucially on the size of the tumor cell population. Low, zero, or negative growth at low population densities is known as the Allee effect; this effect has been studied extensively in ecology, but so far lacks a good explanation in the cancer setting. Here, we formulate and analyze an individual-based model of cancer, in which cell division rates are increased by the local concentration of an autocrine growth factor produced by the cancer cells themselves. We show, analytically and by simulation, that autocrine signaling suffices to cause both strong and weak Allee effects. Whether low cell densities lead to negative (strong effect) or reduced (weak effect) growth rate depends directly on the ratio of cell death to proliferation, and indirectly on cellular dispersal. Our model is consistent with experimental observations from three patient-derived brain tumor cell lines grown at different densities. We propose that further studying and quantifying population-wide feedback, impacting cell growth, will be central for advancing our understanding of cancer dynamics and treatment, potentially exploiting Allee effects for therapy.

## Author summary

A common feature of tumor growth is the production, by the cancer cells themselves, of hormones known as growth factors that increase the rate of cell division. This type of signalling makes the growth rate of the tumor depend on the population size in a non-linear manner, and the growth rate might become low or negative for small population sizes. This is known as the Allee effect which has been studied extensively in ecology. We have developed a computational model that can explain the Allee effect in terms of growth factor signalling, and show by mathematical analysis of the model that the magnitude of the Allee effect depends on the ratio of cell death to proliferation, as well as the properties of the growth factor. In addition we show that the model is consistent with experimental observations from three different cell lines derived from the brain tumor glioblastoma. Our findings indicate that the Allee effect can be exploited in order to improve the treatment of glioblastoma patients.

## 1 Introduction

Cancer growth is increasingly understood as an ecosystem, in which the different cellular components not only grow, but also interact. Such cellular interactions were originally proposed by Laird [1] and the subsequent discovery of growth factors provided a mechanism by which the interactions can occur [2–5]. In the 1970s, Bronk et al [6] showed that dependence on growth factors can give rise to a latent period preceding exponential growth. This suggested that negative feedback at low population density can interfere with exponential tumor expansion. Until recently, however, comparatively little attention has been given to developing mathematical models for these phenomena. The lack of modeling effort may partly reflect the high complexity of cancer cell populations [7], which makes it hard to quantify to what degree functional interactions among cells cause deviations from overall exponential growth [8, 9]. Yet, recent methodological advances make it possible to quantify how interactions between distinct subclones within cell populations affect the growth dynamics of the tumor as a whole [10, 11]. These observations have motivated the formulation of mathematical models that aim to explain nonlinear deviations from exponential growth that occur in cancer cell populations.

One such nonlinear growth behavior, with strong empirical support, is the Allee effect. A central idea in ecological population dynamics, the Allee effect denotes a per-capita growth rate that is reduced at low population densities [12]. There is a distinction between a weak Allee effect, for which the per-capita growth rate increases but remains positive for all densities, and a strong Allee effect where the growth rate becomes negative for sufficiently low densities before approaching zero. In the latter case, there exists a critical population density below which the population will likely go extinct. Therefore, the strong Allee effect has been studied extensively in the context of ecology and species conservation [13]. In theoretical ecology, proposed mechanisms for this include mate limitation (the problem of finding a mate at low population densities), cooperative defense, and cooperative feeding [14–17].

Recently an Allee effect was observed in cancer cell populations cultured in *in vitro* conditions in the lab at limiting densities [18]. Also, strong Allee effects are suggested by *in vivo* xenograft mouse models, where the number of xenotransplanted cancer cells need to exceed a threshold density for a tumor (or metastasis) to form [19]. This threshold depends on the type of tumor cell injected, the host animal strain and site of injection. The possibility of directly observing an Allee effect in human tumors is limited, since the effect is only present at population densities well below the clinical detection threshold at which the tumor typically contains on the order of 10^9^ cells [20]. However, by considering the rate and timing of recurrence after surgery it has been suggested that a weak Allee effect is present among cancer cells that form glioblastomas, a particular form of brain tumor in adults [21]. This conclusion was supported by a mathematical model that describes the tumor growth post-resection, in which the recurrence after surgery was better explained by assuming a weak Allee effect. Further, their results showed that cultured glioblastoma cells indeed exhibited a weak Allee effect.

Autocrine growth factor signaling could be a likely mechanism behind Allee effects in cancer cell populations. Diffusive signaling molecules released by the cancer cells themselves subsequently bind to cell surface receptors, which triggers a signaling cascade ultimately leading to the up-regulation of cell division. In glioblastoma one such growth factor is platelet-derived growth factor (PDGF), which is known to be regularly produced and to up-regulate cell division among glioblastoma cells [22]. This mechanism leads to a type of cooperative behavior and should intuitively lead to an Allee effect.

Here we show that autocrine signaling in an *in vitro* system can give rise to an Allee effect. We study a hybrid individual-based (IB) model that describes the cells as discrete entities and the secreted autocrine growth factor as a continuous field. Further, using analytical tools [23] we derive a mean-field ordinary differential equation (ODE) model for the cell density, which exhibits both a weak and strong Allee effect depending on the ratio of the rates of cell birth to cell death. Lastly, we fit the ODE-model to *in vitro* growth data of glioblastoma cell culture growth and show that an Allee effect is present.

## 2 Methods

### 2.1 Individual-based model

**Fig 1:**
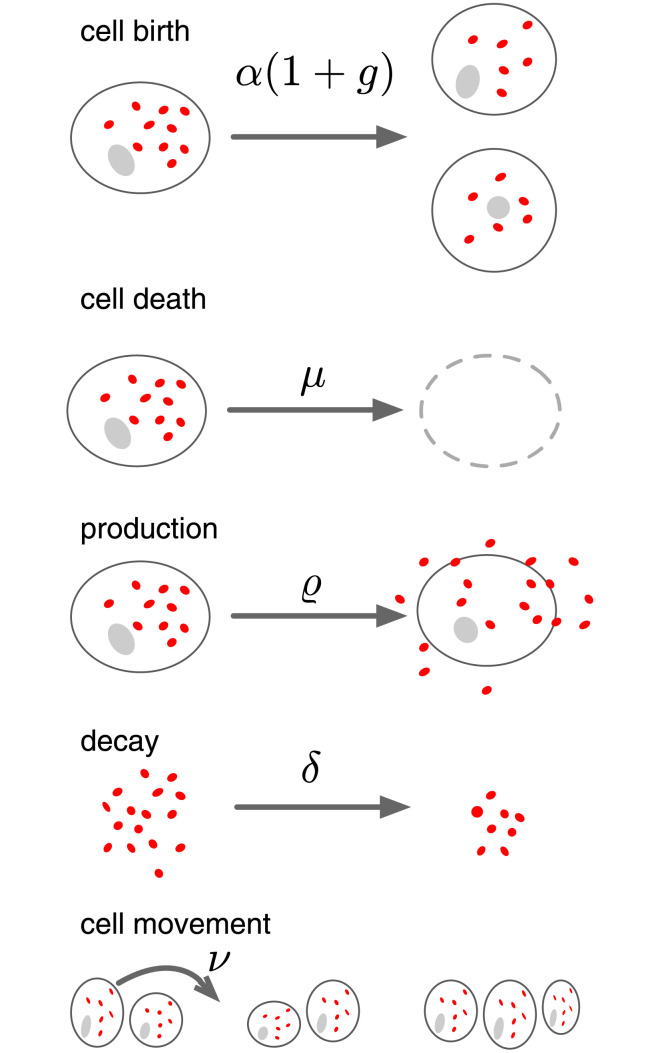
Overview of the mechanisms assumed in the model. Cancer cells divide at a rate *α*(1 + *g*), where *g* is the local growth factor (GF) concentration, and die at a constant rate *μ*. The GF is produced at rate *ρ* by all cancer cells and decays at rate *δ*. Lastly, cells migrate at rate *ν*. Parameter values are provided in Table 1.

In order to model the effects of autocrine signaling we consider an individual-based (IB)-model in which the cells reside on a two-dimensional square lattice (see Fig 1(a) and [23] for details). The linear size of the domain is *L* = .2 cm and it contains *N* × *N* lattice sites, each with a diameter *d* = *L*/*N*. For cancer cells a reasonable value is *N* = 100, which gives a cell size of *d* = 20 *μ*m [24]. The growth factor (GF) concentration evolves according to

$$\frac{\partial g(\vec{x},t)}{\partial t}=D\nabla^{2}g\left(\vec{x},t\right)+\rho \;c\left(\vec{x},t\right)-\delta \;g\left(\vec{x},t\right)$$ (1)

where $c(\vec{x},t)=1$ at all sites that are occupied by cells and zero otherwise. The growth factor diffuses with diffusion constant *D*, is produced at rate *ρ* and decays at rate *δ*. The partial differential equation is subject to no-flux boundary conditions, representing a closed experimental system.

The cell population changes in the following way: A cell located at site $\vec{x}$ divides at a rate

$$\begin{array}{c} \lambda =\alpha (1+g\left(\vec{x},t\right)), \end{array}$$ (2)

where $g(\vec{x},t)$ is the local GF-concentration. We consider two different modes of dispersal upon cell division. For long range dispersal the daughter cell is placed uniformly at random among all sites on the lattice, whereas for short range dispersal the daughter is placed uniformly at random among neighbouring lattice sites, using a von Neumann neighborhood. In both cases, if the chosen site is occupied cell division fails. Cells are assumed to die at a constant rate *μ*, and also move into empty (von Neumann) neighboring sites at rate *ν*. Movement occurs at random into neighboring sites and fails if the target site is occupied. An overview of the model is shown in Fig 1. All parameters are given in Table 1. Since the model is general we chose parameter values related to the growth factor that give rise to noticeable levels of autocrine signalling.

**Table 1:** Model parameters.

*α*	Baseline cell division rate	10^−5^ [25]	s^−1^
*μ*	Rate of cell death	10^−6^ − 10^−4^ [26]	s^−1^
*δ*	Growth factor decay rate	10^−3^ [-]	s^−1^
*ρ*	Growth factor production rate	10^−2^ [-]	s^−1^
*D*	Growth factor diffusion coefficient	5 × 10^−9^ [27]	cm^2^/s
*D*_*c*_	Cellular diffusion coefficient	2 × 10^−10^ (obtained from data)	cm^2^/s
*L*	Size of the domain	0.2 [-]	cm
*d*	Cellular diameter	20 [24]	*μ*m

### 2.2 Analytical results

In order to understand the dynamics of the IB-model we employ a technique developed by Gerlee & Altrock [23], which makes it possible to derive an ordinary differential equation for the density of cells. This derivation is only exact in the case of long range dispersal, but we will also compare the analytical results with dynamics of short range dispersal. The main idea behind the method is to represent the stochastic distribution of cells in the IB-model as a Fourier series and from that compute the expected growth rate. Previously, the method was developed to describe a situation where the population consists of two distinct subpopulations: producers that produce a diffusible public good that is costly to produce and free-riders that are identical to producers except they do not produce the public good and consequently do not pay the corresponding reproductive cost.

In order to adapt the model to the case of a homogeneous population where all cells produce the public good we absorb the cost of the public good into the baseline division rate. The equation describing the population size is given by (see [23] for details of the derivation):

$$\frac{dn(t)}{dt}=f(n)=\Gamma (n)n(1-n)-\mu n,$$ (3)

where *n*(*t*) is normalized with respect to the carrying capacity (the maximal population size *N*^2^) and therefore ranges from 0 to 1, and the division rate Γ(*n*) is density-dependent and given by

$$\begin{array}{c} \Gamma \left(n\right)=\alpha +n\frac{\alpha \rho }{\delta }+\frac{\alpha \rho K}{N}(1-n). \end{array}$$ (4)

The different terms in the growth rate Γ(*n*) can be given distinct interpretations: the first term is the baseline growth rate in the absence of the GF, the second term is the average GF-contribution from all cells and the last term is an additional growth benefit from the GF due to increased local GF concentration, which is larger for low densities when the factor 1 − *n* is large. This quantity depends on

$$\begin{array}{c} K=\frac{1}{2\delta }+\frac{L}{4\sqrt{\delta D}}, \end{array}$$ (5)

and scales with a factor 1/*N* = *d*/*L* = 0.01 in (4), implying that as the cell size *d* decreases (in relation to the system size *L*) the direct benefit is reduced.

### 2.3 Experimental methods

Cells from the cell lines U3013MG, U3123MG and U3289MG obtained from the Human Glioma Cell Culture (HGCC) resource [28] were suspended in serum-free neural stem cell (NSC) medium, supplemented with B-27, N2, EGF, FGF and plated on 384 well plates (BD Falcon Optilux TC #353962) coated in laminin. Six different initial densities were used ranging from 125–4000 cells/well and each density was replicated eight times. The cells were cultured at 37°C and 5% CO_2_ for 120 hours and imaged using an IncuCyte microscope at 20x magnification every 15–20 minutes (the exact time varied throughout experiments). The images were segmented using Fogbank [29] (see S1 Supplementary Methods for details). The normalized cell density (degree of confluency) was estimated by calculating the ratio of the total number pixels belonging to cells to the total number of pixels in the image. Growth curves for each initial density were calculated by averaging the normalized cell density across all eight replicates. In order to avoid seeding effects the images collected during the 3.5 first hours were discarded. In order to make sure that the confluency is a good proxy for cell density (i.e. cell number per well) we counted the number of cells per image and calculated the correlation coefficient between the cell count and confluency. The average correlation coefficient across all wells was 0.96, 0.88 and 0.92 for the three cell lines suggesting that confluency is indeed a good proxy. See Fig A in S1 Supplementary Methods for a visual comparison of the cell count and confluency for a single well. In order to estimate the rate of migration of the cell lines we calculated the diffusion coefficients in the following way: images from all wells were segmented as described above and the Trackpy tracking algorithm was used in order to obtain individual cell tracks [30] (see SI for details). All tracks were centred at the origin and shifted to *t* = 0. The mean squared displacement (MSD) was calculated according to

$$\begin{array}{c} \text{MSD}\left(t_{k}\right)=\frac{1}{N}\sum_{i=1}^{N}{\left|{\text{x}}^{\left(\text{i}\right)}\left(t_{k}\right)\right|}^{2}, \end{array}$$

where *t*_*k*_ correspond to the *k*^th^ time point, and *N* is the number of trajectories. Due to cells leaving and entering the field of view we discarded all data points beyond 10 hours. We fitted a line to the MSD using least squares regression and used the relation MSD(*t*) = 4*Dt*, where *D* is the diffusion coefficient. To obtain an estimate of the diffusion coefficient for each cell line we averaged the result over all initial densities and replicates. Fig B in S1 Supplementary Methods shows an example of the MSD and the linear regression.

The parameters of the ODE-model (3) were estimated by minimising the squared error between the model and the data for all growth curves simulatenously using the error function:

$$\begin{array}{c} E\left(\theta \right)=\sum_{m=1}^{6}\sqrt{\frac{1}{k}\sum_{i=0}^{k}(n\left(t_{i},\theta \right)-N_{m}\left(t_{i}\right){)}^{2}}, \end{array}$$ (6)

where *k* is the number of time points *t*_*i*_ and the outer sum runs across the growth curves obtained for different initial densities. The numerical solution of the ODE-model *n*(*t*, *θ*) was obtained by using the normalized density at *t* = 0 as the initial condition and *θ* = (*A*, *B*, *μ*) are the parameters of the model. Numerical solutions were calculated using an Euler-forward scheme with time step of 0.25 hours.

## 3 Results

First, we present results obtained from analyzing an ordinary differential equation (ODE)-model of the system (3). Next, we test the validity of these results by comparing them to outcomes from our individual-based simulation approach. Last, to confirm our theoretical predictions, we analyze experimental results by fitting the ODE-model to time series data of *in vitro* cultured glioblastoma cells.

### 3.1 Analysis of the ODE-model

**Fig 2:**
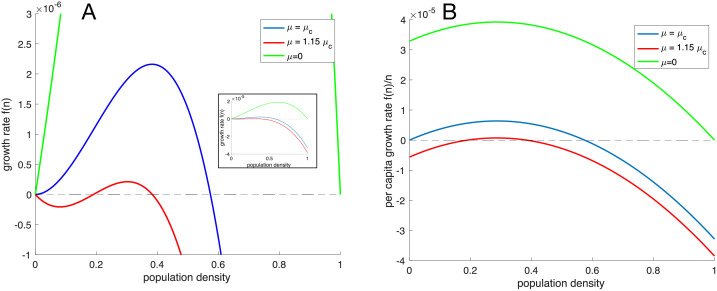
Growth rates. (A) The population growth rate (3) as a function of the population density for three different values of the death rate *μ*. The inset shows the entire range of growth rates. (B) The per-capita growth rate *f*(*n*)/*n* as a function of the population density for three different values of the death rate *μ*. Here the Allee effect is evident as an increasing per-capita growth rate at low densities. All parameter values are given in Table 1 and the critical death rate is calculated according to (8).

Depending on the relation between the parameters in the ODE-model (3) it can give rise to different dynamics. We will here focus on the impact of the baseline division rate *α* and the death rate *μ*. For *μ* = 0 the system (3) has two non-negative fixed points, *n*^⋆^ = 0 and *n*^⋆^ = 1 (see Fig 2A), corresponding to a system void of cells and at carrying capacity respectively. This holds true as long as Γ(*n*) = 0 has no positive solutions, which is the case for the baseline parameters. The presence of a density-dependent division rate leads to non-monotonous per-capita growth rate (*f*(*n*)/*n*) as can be seen in Fig 2B. This is in contrast with the case where Γ(*n*) = constant (i.e. logistic growth) where the per-capita growth rate equals 1 − *n* − *μ* and is a linear and decreasing function of *n*.

For small values of *μ*, the per-capita growth rate is an increasing function for small densities, but remains positive. This is known as a weak Allee effect [13], whereas for higher *μ* the per-capita growth rate becomes negative for small densities leading to the extinction of population below a certain critical threshold, which is termed a strong Allee effect [13] (see Fig 2B). The existence of a strong Allee effect is thus equivalent to the fixed point at the origin *n*^⋆^ = 0 being stable rather than unstable. The stability can be determined using linear stability analysis and the criterion for stability is that *f*′(0) > 0. We find that the fixed point at *n* = 0 is stable (or equivalently a strong Allee effect exists) when

$$\begin{array}{c} \alpha +\frac{\alpha \rho Kd}{N}<\mu . \end{array}$$ (7)

The critical death rate above which we expect to observe a strong Allee effect in the IB-model is thus given by

$$\begin{array}{c} \mu_{c}=\alpha +\frac{\alpha \rho Kd}{N}. \end{array}$$ (8)

The critical density *n*_*c*_ below which the population is driven to extinction can be calculated explicitly as the unstable interior fixed point of (3), and is given by

$$\begin{array}{c} n_{c}=\frac{N(\rho -\delta )-2\rho \delta K}{\rho (N-\delta K)}-\sqrt{(\frac{N(\rho -\delta )-2\rho \delta K}{\rho (N-\delta K)}{)}^{2}-\frac{\mu \delta N}{\rho N-\rho \delta K}}. \end{array}$$ (9)

### 3.2 Comparison to the IB-model

We now move onto comparing our analytical predictions to the IB-model.

#### 3.2.1 Long range dispersal

**Fig 3:**
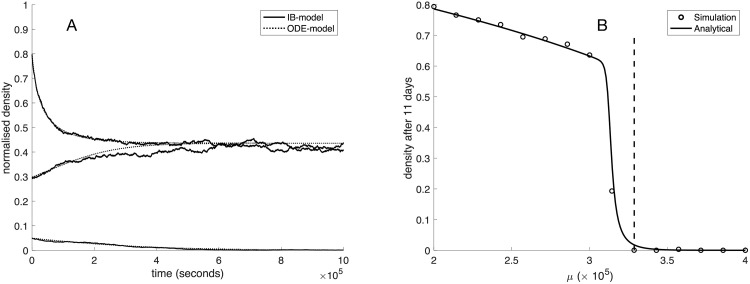
Comparison of the ODE-model and IB-model under long range dispersal. (A) The dynamics of the IB-model (dashed) and the numerical solution of (3) (solid) under long range dispersal for three different initial conditions. All parameter values are given in Table 1 and the death rate *μ* = 3.75 × 10^−5^. (B) The population density after 11 days of the IB-model (circles) and the numerical solution of (3) (solid line) under long range dispersal and no cell migration when the death rate *μ* is varied. All other parameter values are given in Table 1.

We start by looking at the case when dispersal is long range and newborn cells are dispersed randomly throughout the entire domain. Fig 3A shows a comparison between the IB-model and a numerical solution of the ODE-model (3) for three different initial conditions using the baseline parameter values (see Table 1). Agreement between the IB-model and the ODE is very good and we can conclude that in this scenario autocrine signaling induces a strong Allee effect, since low initial densities give rise to population extinction.

In order to investigate the impact of the death rate on the Allee effect we initialized the system at a low initial population size of *n*_0_ = 10^−2^ for death rates in the range 2 − 4 × 10^−5^ and ran the IB-model for 11 days and recorded the density of cells. This was compared to the numerical solution of the ODE-model and the result is shown in Fig 3B, where the vertical dashed line corresponds to the theoretically predicted death rate *μ*_*c*_ at which the strong Allee effect emerges. Please note that this value will deviate slightly from the results of the IB-model since the simulations are initialized with small, but non-zero density. A more exact value of the critical death rate can be obtained by setting *n*_*c*_ = *n*_0_ = 10^−2^ in (9) and solving for the death rate *μ*. This expression is however much more complicated than the simple expression for the critical death rate (8).

#### 3.2.2 Short range dispersal

**Fig 4:**
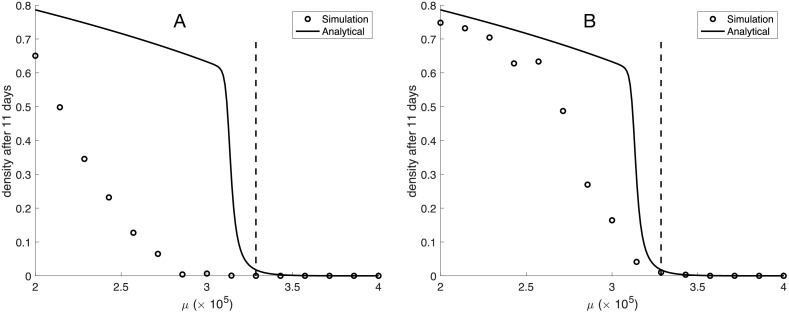
The population density after 11 days as a function of the death rate *μ*. Results from the IB-model (circles) and the numerical solution of (3) (solid line) under (A) short range dispersal and no cell migration and (B) short range dispersal and a cellular diffusion coefficient of *D*_*c*_ = 1.6 × 10^−10^ cm^2^/s. All other parameter values are given in Table 1.

Let us now analyze the case when newborn cells are placed next to the parent cell. In this scenario cell migration rate becomes an important parameter since movement of cells tends to reduce spatial correlations and bring the system closer to the mean-field limit. We therefore start by analyzing the extreme case of short range dispersal and no cell migration, and again compare the long-term dynamics of the IB-model with the numerical solution of the ODE-model (3). By comparing the long-term dynamics we see that the ODE-model severely over-estimates both the population density and the critical death rate *μ*_*c*_ (see Fig 4A). This might seem surprising since local dispersal leads to clumping of cells, and cells in such a configuration would, on average, experience a higher GF-concentration (compared to an evenly dispersed population) due to the production from neighbouring cells. However, local dispersal also leads to increased competition for space and therefore has a negative effect on the rate of division. This negative effect emerges because cells that are trapped by neighbouring cells cannot divide, which reduces the effective birth rate. This latter effect seems to dominate for the baseline parameter values. As a result the Allee effect becomes even more pronounced.

Of note, we here approach cellular dispersal in the IB-model exclusively. An explicit incorporation of space in an analytical model formulation would lead to a partial differential equation (PDE) that, in turn, could result in an ODE if space is integrated out. Comparison between IB, PDE, and ODE approaches exist [31], which become particularly interesting in settings where computational efficiency is crucial.

We sought to determine whether our experimental cell culture system exhibits migration rates that reduce spatial effects and renders the ODE-model a valid description. Thus, we measured the mean squared displacement (MSD) of each cell line, and found that the cellular diffusion coefficients for U3013MG, U3123MG and U3289MG were given by 1.82 × 10^−10^, 1.67 × 10^−10^ and 1.31 × 10^−10^ cm^2^/s. Using a cellular diffusion coefficient given by the average *D*_*c*_ = 1.6 × 10^−10^ cm^2^/s in the IB-model ameliorated the spatial effects and improves the agreement between the IB-model and the analytical result (see Fig 4B).

### 3.3 Comparison to experimental data

Having ascertained that the ODE-model gives an accurate description of the IB-model at realistic rates of cell migration we now move on to fitting the ODE-model to experimental data. We assume a growth rate of the form

f(n)=(A+Bn)n(1-n)-μn, (10)

where

$$A=\alpha +\frac{\alpha \rho K}{N}$$ (11)

$$B=\frac{\alpha \rho }{\delta }-\frac{\alpha \rho K}{N}.$$ (12)

**Fig 5:**
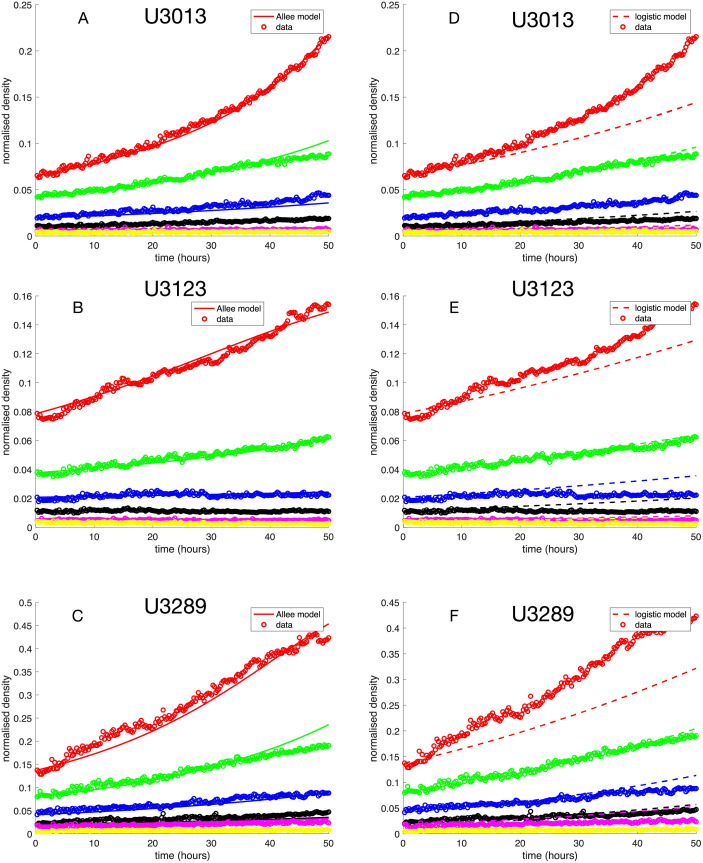
Least squares fit of the Allee ODE-models to *in vitro* growth of the glioblastoma cell lines U3013MG, U3123MG and U3289MG. Panels A-C show the best fit of the Allee model whereas D-F show the best fit for a logistic growth model (where *ρ* = 0). Visual inspection suggests that the Allee model outperforms the logistic model. This was confirmed using Akaike Information Criterion (see Table 2).

We used least squares minimisation to find numerical values of the constants *A*, *B* and *μ* for each cell line such that the deviation between the model and growth curves for six different initial population sizes was minimised (6). The optimal parameters for each cell line can be found in Table 2 and a comparison between the growth curves and the model dynamics can be seen in Fig 5A–5C.

**Table 2:** Details of model fit to data. The parameter values (*A*, *B*, *μ*) refer to the optimal fit for the Allee model. For a visual comparison see Fig 5.

Cell line	*AIC*_Allee_	*AIC*_logistic_	*A*	*B*	*μ*	Type
3013	−3.96 × 10^3^	−2.76 × 10^3^	0.082	0.254	0.074	Weak
3123	−4.52 × 10^3^	−3.13 × 10^3^	1.787	2.202	1.792	Strong
3289	−2.48 × 10^3^	−1.6 × 10^3^	0.131	0.274	0.123	Weak

In order to investigate if the data could be explained by a simpler model we also fitted a standard logistic growth function, which corresponds to the special case of zero GF production (*ρ* = 0). The logistic model only has two parameters: a birth rate and a death rate. We found that the logistic model gave larger model error across all cell lines.

In order to account for the larger number of parameters in the ODE-model we also calculated the Akaike information criterion (AIC) given by

$$\begin{array}{c} AIC=2k+n\;\text{ln}(\text{RMSE}), \end{array}$$

where *k* is the number of model parameters, *n* is the number of data points and RMSE is the model error (6) [32]. We found that the ODE-model with an Allee effect has a lower AIC compared to the logistic model for all cell lines (see Table 2). We thus conclude that the ODE-model is a better description of the experimental data compared to the logistic equation.

When comparing the optimal parameters for the cell lines we note that for U3013MG and U3289MG we have *A* > *μ* implying a weak Allee effect, whereas for U3123MG we have *μ* > *A* which corresponds to a strong Allee effect. These differences can also been seen in Fig 5B where low initial densities lead to a declining population. Of note, there could be lack of precision in the non-normalised population density’s maximum values due to a focus on data describing early time, low-density dynamics [33, 34]. Attempts to alleviate this discrepancy [33] connect parameter estimation, model selection and experimental design in a deeper way, to potentially describe both low and high (near carrying capacity) density dynamics.

## 4 Discussion

We have proposed a mathematical model to explain the Allee effect, a phenomenon observed in many experimental datasets of cancer cell population dynamics. Our model, which posits that autocrine secreted growth factors increase the rate of cell division, yields population dynamics that can exhibit a weak or strong Allee effect depending on the relationships between the model parameters. Fitting the model to a dataset of three patient-derived glioma cell lines showed that a parameter setting with an Allee effect provided the best fit.

Due to the large heterogeneity between patient-derived cell lines coupled to the experimental difficulties of measuring growth factor production and decay, we chose an approach where we used the IB-model as a means of showing that an Allee effects is possible. This observation was confirmed by our experimental data, but parametrizations of individual experiments remain difficult. Additional experiments to measure, e.g. the rate of growth factor production and its precise impact on cell division would be needed, as these two parameters are not identifiable in the ODE model. The situation is further complicated by the fact that several distinct growth factors have been identified as autocrine signals in glioblastoma, e.g. TGF-*α* and EGF [35].

In recent years many studies have focused on the Allee effect in cancer, either in an effort to understand its origin or to investigate how the effect can be therapeutically exploited [36]. For example, Böttger et al. [37] linked plasticity to an Allee effect in glioblastoma. Another potential cause of the effect was proposed by Konstorum et al. [38], who investigated the impact of feedback regulation in cancer stem cell dynamics. The impact of density-dependent proliferation rates was explored by Johnson et al. [18], and similarly by Fadai et al. [39], who both connected the per-capita growth rates in an individual-based model with coefficients in an ODE-model that recapitulated the growth rate decline as cell population sizes decrease.

Neither of the above mentioned results were directly linked to production and consumption of secreted factors. Böttger et al. [37] approached the dynamics using an agent based approach, and studied switching between migratory and proliferation, triggered by the microenvironment in the form of local cell density. Fadai et al. [39] modeled local-level binary switches stochastically, and showed that these dynamics can lead to a family of Allee effects. Johnson et al. [18] focused on modeling Allee effects together with spatial invasion and showed that these systems can exhibit shock-fronted travelling wave scenarios. These models have adressed interesting connections between stochasticity, spatial invasion and patterns in the tumor microenvironment, but did not address the *emergence* of Allee effects based on a feasible bio-physical mechanism such as secretion and uptake of growth-stimulating molecules. Thus, our integrative framework here complements these results by directly showing that autocrine signalling induced, and density-dependent feedback, can have detrimental effects on the per-capita growth rate of the cell population. We showed that this type of interaction is plausible to generate an Alee effect, integrating an analytical framework with agent based modeling and novel experimental results.

We provide a mechanistic explanation for a density-dependent proliferation rate in terms of local growth factor concentration. Mathematical analysis of the individual-based model allowed for the derivation of an ODE-model whose coefficients depend on the IB-model parameters. Our ODE-model was fitted to experimental data, and we found that a model which included an Allee effect best explained the empirical observations, in accordance with a previously observed weak Allee effect in breast cancer cell populations [39]. These and our findings lead to the hypothesis that a more general pattern of self-interaction-driven negative feedback among cell lines in which autocrine signaling could be present. However, it should be noted that our results do not provide conclusive evidence as to the origin of the Allee effect in the experimental data. This would require investigating the possible pathways of autocrine signalling (e.g. PDGF, TGF-*β*, EGF) and is beyond the scope of this paper.

Another way of testing the hypothesis put forth is to investigate the impact of local cell density on the rate of cell division on the cell lines considered in this study. Given microscopy time-lapse data it should possible to estimate the impact of neighbouring cells on the rate of division. An inferred rate of division as an increasing function of the number of neighbouring cells would lend strong support to a localised interaction (e.g. a diffusing GF) as the potential origin of the Allee effect.

In light of cancer therapy, a strong Allee effect would be more beneficial for the ability to control a tumor, since the strong effect leads to population extinction at critically low population densities. Such conditions are typically present after therapeutic interventions (e.g. surgery and radiotherapy). If the population dynamics could be tipped towards a strong Allee effect during therapy, similar to an extinction threshold [40], one could observe decreased risk of recurrence. This critical decline could for example be achieved by reducing the effect of the growth factor by inhibiting its binding to cell surface receptors, or by increasing the factor’s decay rate. Beyond these threshold considerations, the presence of Allee effects has implications for tumor control that needs to consider the emergence of aggressive variants that were previously below detectable thresholds. These variants could receive an advantage, as the previously dominant cancer cell population against which treatment is initially chosen observes fitness decreases due to an Allee effect.

In conclusion, our findings provide a general model for the population dynamics of cancer cells driven by autocrine signaling. We provide a possible mechanistic explanation for the ubiquitous Allee effect. Further, our findings warrant more research into the therapeutic benefits of altering the effects of autocrine signaling to understand and achieve tumor control.

## Supporting information

S1 Supplementary Methods Processing of image data: This text describes the image processing pipeline including settings for FogBank and PyTrack. **Fig A**: The confluence plotted against the cell count for a single well across all time points. **Fig B**: The mean squared displacement as a function of time averaged across all cells in a single well. The dashed line shows the best fit for a line with intercept *y* = 0. The slope of the line equals 4*D*, where *D* is the diffusion coefficient of the cells.
(PDF)
