# Supplementary material for: Autocrine signaling can explain the emergence of Allee effects in cancer cell populations
Source: PLoS Comput Biol. 2022 Mar 3;18(3):e1009844. doi: 10.1371/journal.pcbi.1009844 (PMC8923455; doi:10.1371/journal.pcbi.1009844)
Supplement: S1 Supplementary Methods — Fig A: The confluence plotted against the cell count for a single well across all time points. Fig B: The mean squared displacement as a function of time averaged across all cells in a single well. The dashed line shows the best fit for a line with intercept y = 0. The slope of the line equals 4D, where D is the diffusion coefficient of the cells. (PDF) [file pcbi.1009844.s001.pdf]

Supporting information for “Autocrine signaling explains the emergence of Allee effects in cancer cell populations”

Supporting Methods: Processing of image data

The following steps have been taken in order to analyze the cell images to obtain migration tracks.

- Adjust contrast using MATLABs built-in function **imadjust**, with settings low_in = 0.3, high_in = 0.7, low_out = 0, high_out = 1.
- Apply a tophat filter using MATLABs built-in function **imtophat** on the contrast adjusted image, with a disk-shaped structuring element of radius 4 using **strel('disk',4)**.
- Use FogBank with default settings, except the following setting changes. Min cell area = 100, Morphological operation = erode with radius 0. In the object separation section the following settings were used. Min Seed Size = 250, FogBank direction = Max $\rightarrow$ Min. Min object area = 100.
- The FogBank labelled masks were converted into binary images for improved tracking performance. This was done by computing the center of mass of each labelled cell, and replaced with a square shape of size 7×7 pixels.
- Trackpy was used on the binarized images to obtain migration trajectories. The featuresize was set to 15, minmass to 5000. During the linking phase the maximum displacement was set to 20, and memory to 3. The stub filter length was set to 4.

Supporting figures

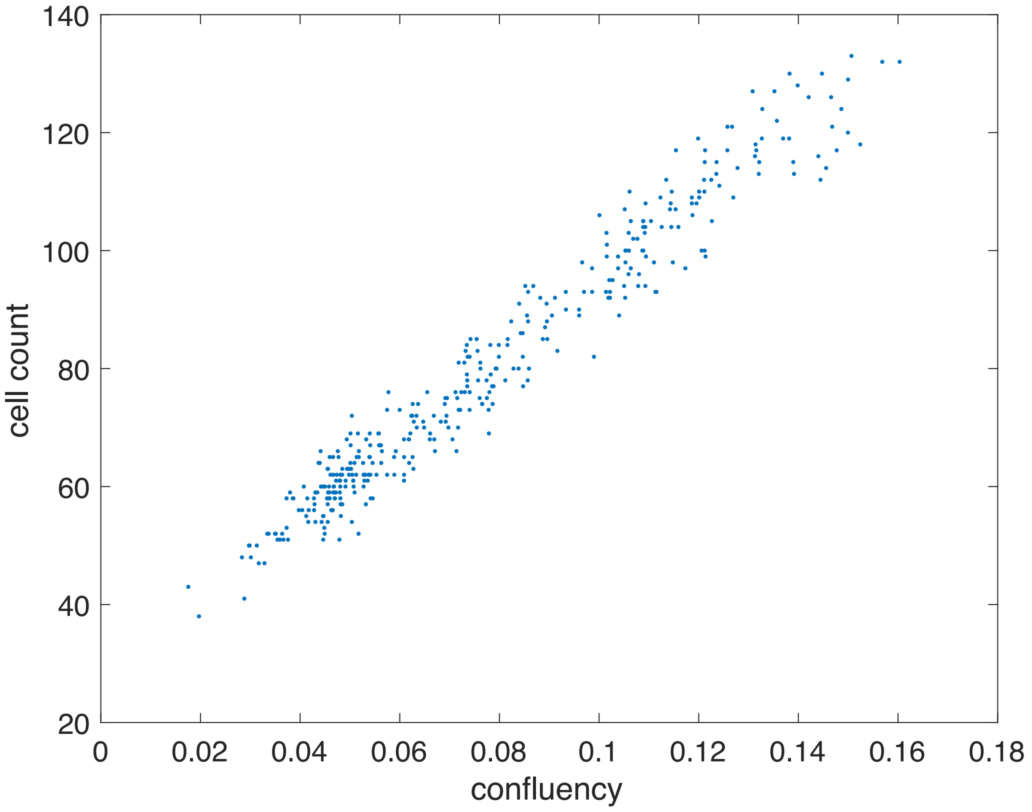

Figure A: The confluence plotted against the cell count for a single well across all time points.

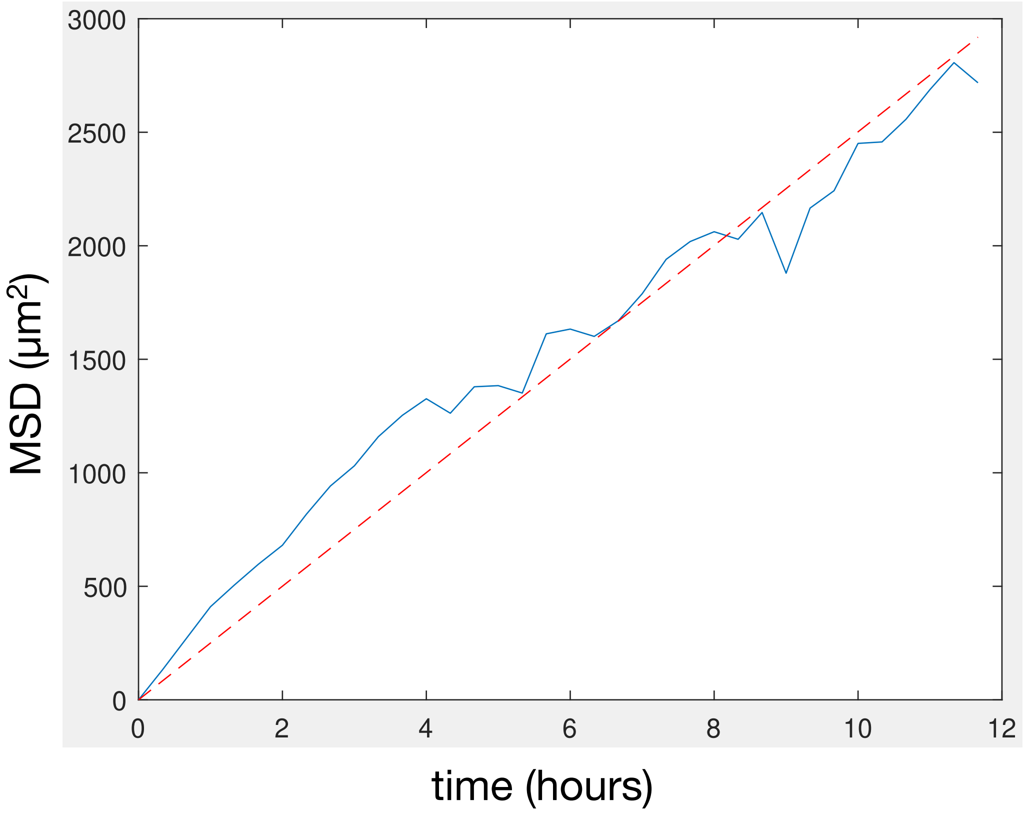

Figure B: The mean squared displacement as a function of time averaged across all cells in a single well. The dashed line shows the best fit for a line with intercept $y = 0$. The slope of the line equals $4D$, where D is the diffusion coefficient of the cells.
